# A de novo frameshift variant of *ANKRD11* (c.1366_1367dup) in a Chinese patient with KBG syndrome

**DOI:** 10.1186/s12920-021-00920-3

**Published:** 2021-03-02

**Authors:** Jing Chen, Zhongmin Xia, Yulin Zhou, Xiaomin Ma, Xudong Wang, Qiwei Guo

**Affiliations:** 1 United Diagnostic and Research Center for Clinical Genetics, Women and Children’s Hospital, School of Medicine and School of Public Health, Xiamen University, Xiamen, 361102 Fujian China; 2 Department of Child Health, Women and Children’s Hospital, School of Medicine, Xiamen University, Xiamen, 361102 Fujian China; 3 Department of Radiology, Women and Children’s Hospital, School of Medicine, Xiamen University, Xiamen, 361102 Fujian China

**Keywords:** KBG syndrome, *ANKRD11*, Frameshift variant, Physical therapy, Whole-exome sequencing, Case report

## Abstract

**Background:**

KBG syndrome is a rare autosomal dominant genetic disease mainly caused by pathogenic variants of *ankyrin repeat domain-containing protein 11 (ANKRD11)* or deletions involving *ANKRD11*. Herein, we report a novel de novo heterozygous frameshift *ANKRD11* variant via whole exome sequencing in a Chinese girl with KBG syndrome.

**Case presentation:**

A 2-year-2-month-old girl presented with a short stature and developmental delay. Comprehensive physical examinations, endocrine laboratory tests and imaging examination were performed. Whole‐exome sequencing and Sanger sequencing were used to detect and confirm the variant associated with KBG in this patient, respectively. The pathogenicity of the variant was further predicted by several in silico prediction tools. The patient was diagnosed as KBG syndrome with a short stature and developmental delay, as well as characteristic craniofacial abnormalities, including a triangular face, long philtrum, wide eyebrows, a broad nasal bridge, prominent and protruding ears, macrodontia of the upper central incisors, dental crowding, and binocular refractive error. Her skeletal anomalies included brachydactyly, fifth finger clinodactyly, and left-skewed caudal vertebrae. Electroencephalographic results generally showed normal background activity with sporadic spikes and slow wave complexes, as well as multiple spikes and slow wave complexes in the bilateral parietal, occipital, and posterior temporal regions during non-rapid-eye-movement sleep. Brain MRI showed a distended change in the bilateral ventricles and third ventricle, as well as malformation of the sixth ventricle. Whole exome sequencing revealed a novel heterozygous frameshift variant in the patient, *ANKRD11* c.1366_1367dup, which was predicted to be pathogenic through in silico analysis. The patient had received physical therapy since 4 months of age, and improvement of gross motor dysfunction was evident.

**Conclusions:**

The results of this study expand the spectrum of *ANKRD11* variants in KBG patients and provide clinical phenotypic data for KBG syndrome at an early age. Our study also demonstrates that whole exome sequencing is an effective method for the diagnosis of rare genetic disorders.

**Supplementary Information:**

The online version contains supplementary material available at 10.1186/s12920-021-00920-3.

## Background

KBG syndrome (MIM # 148050) is a rare autosomal dominant genetic disease that was first described by Herrmann in 1975 [1]. The typical phenotypes include intellectual disability, developmental delay, macrodontia of the upper central incisors, characteristic craniofacial features, hearing loss, skeletal abnormalities, and short stature [2–6]. The prevalence of this disease remains unknown. To date, more than 200 patients with KBG syndrome have been reported worldwide [7]. However, it is likely that this syndrome is underdiagnosed, since many of the features are mild and do not specific to this syndrome [2]. The genetic causes of KBG syndrome include single nucleotide variants and small indels of *ANKRD11* or microdeletions of 16q24.3 involving *ANKRD11*, accounting for approximately 83% and 17% of cases, respectively [8–12]. *ANKRD11* encodes ankyrin repeat domain-containing protein 11, an ankyrin repeat domain-containing cofactor that plays essential roles in embryogenesis, skeletogenesis, and bone turnover [13, 14]. To date, more than 300 variants in *ANKRD11* have been identified in cases of KBG syndrome according to ClinVar database, and the majority of these variants occurred de novo [8].

In this study, we report the analysis of a de novo heterozygous frameshift *ANKRD11* variant in a Chinese girl with KBG syndrome and the corresponding phenotypes.

## Case presentation

**Fig. 1:**
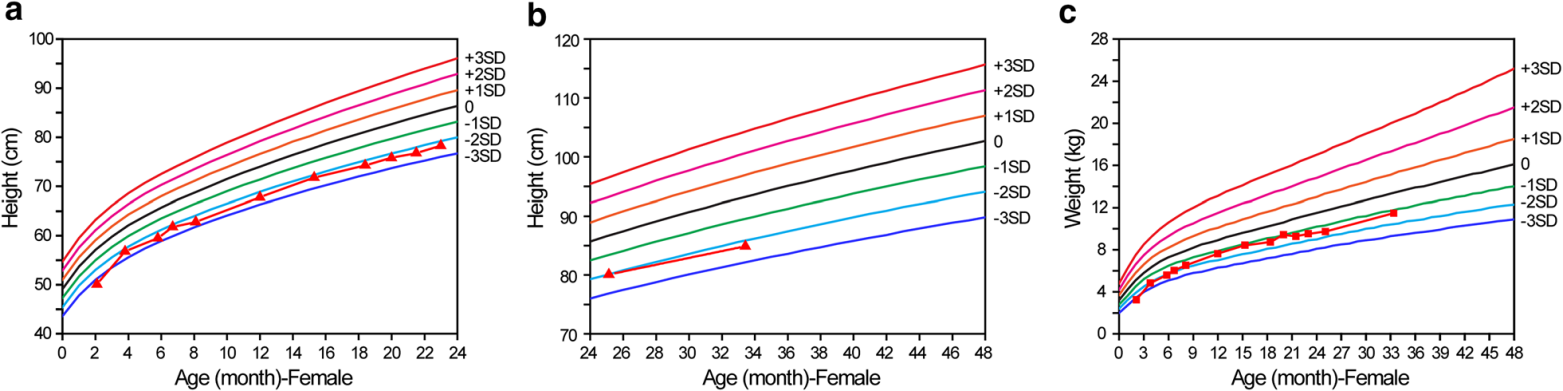
Evaluation of height and weight of the patient. **a** Height of patient at the age period between 0 and 24 months (measured in lying down state). **b** Height of patient at the age period between 24 and 34 months (measured in standing state). **c** Weight of patient. The triangles and squares indicate the height and weight measurements of the patient, respectively

The patient was a Chinese girl who was born at 37^+3^ weeks gestation via spontaneous vaginal delivery after an uneventful pregnancy. Her birth length and weight were 45.0 cm (− 2.2 SD) and 2200 g (− 2.5 SD) respectively, and her head circumference was 31 cm (− 2.4 SD). The height of her mother and father were 168 cm and 155 cm, respectively. After birth, the patient showed feeding difficulty, short stature (Fig. 1), motor dysfunction, and developmental delay (Table 1). She began to walk and talk at 17 and 25 months, respectively. No hearing or behavioral issue was noted.

**Table 1:** Neuropsychological evaluation of the patient

Age (M)	Gross motor		Fine motor		Adaptive strengths		Language		Social behaviors		General evaluation	
	DQ	MA	DQ	MA	DQ	MA	DQ	MA	DQ	MA	DQ	MA
15.3	65	10.0	65	10.0	72	11.0	59	9.0	72	11.0	67	10.2
21.0	93	19.5	71	15.0	71	15.0	71	15.0	57	12.0	73	15.3
25.6	94	24.0	70	18.0	64	16.5	74	19.0	59	15.0	72	18.5
33.4	90	30.0	76	25.5	72	24.0	67	22.5	76	25.5	76	25.5
37.6	96	36.0	76	28.5	76	28.5	60	22.5	76	28.5	77	28.8

DQ = MA/M × 100%. DQ ≥ 130: exellent; DQ = 115–129: good; DQ = 85–114: average; DQ = 70–84: intermediate; DQ ≤ 70: poor

*M* month, *DQ* developmental quotient, *MA* mental age

**Fig. 2:**
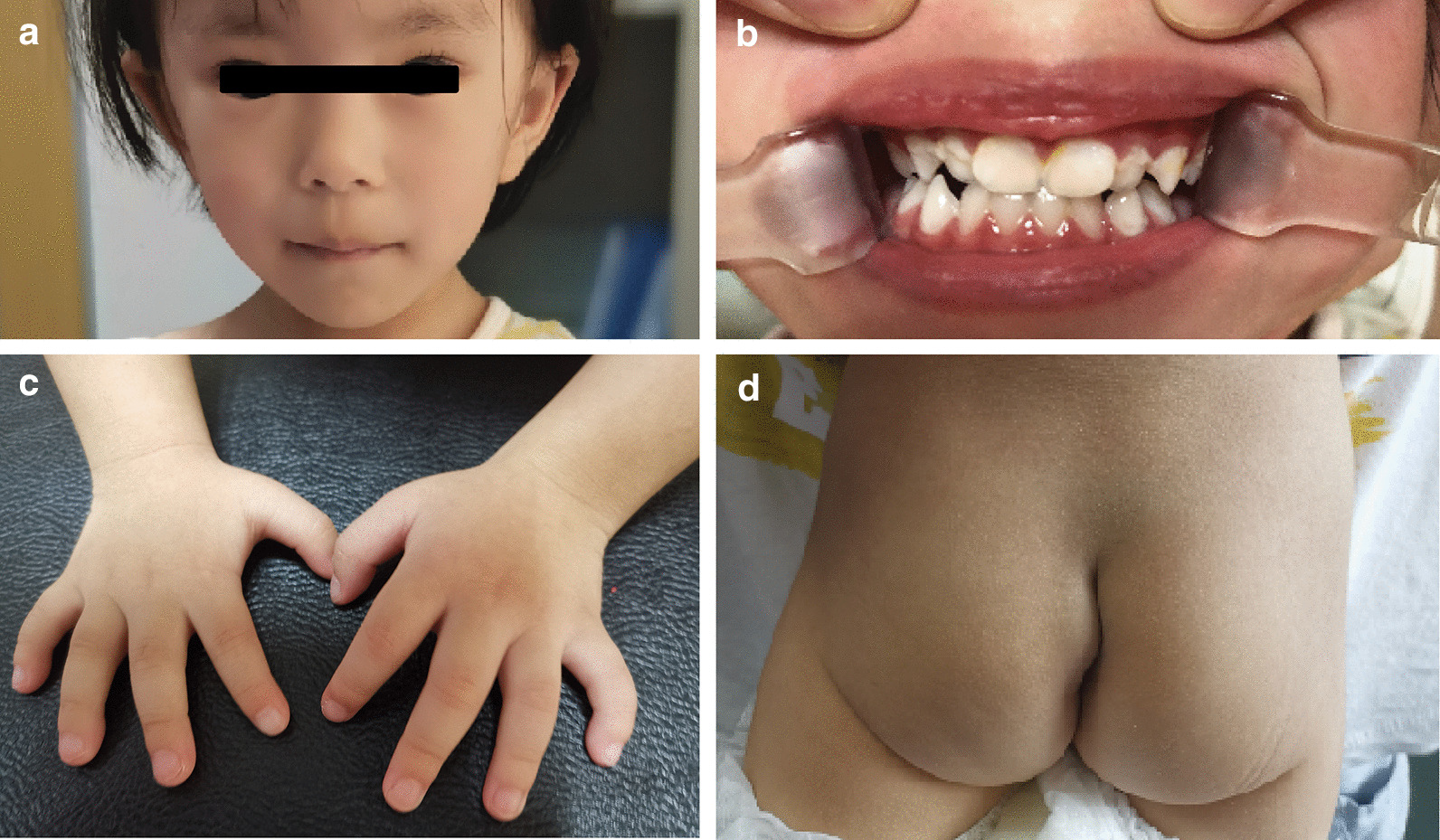
Physical examination of the patient. **a** Craniofacial appearances. **b** Macrodontia of upper central incisors (dental crown: 7.0 mm × 7.7 mm) and dental crowding. **c** Fifth fingers clinodactyly. **d** Abnormal caudal vertebra shape

At the age of 2 years and 2 months, she received a comprehensive physical examination, which showed some suspect craniofacial features, including a triangular face, long philtrum, wide eyebrows, broad nasal bridge, prominent and protruding ears, macrodontia of the upper central incisors (dental crown: 7.0 mm × 7.7 mm), dental crowding, and binocular refractive error (Fig. 2). Other physical examination findings included brachydactyly, fifth finger clinodactyly, delayed anterior fontanel closure, and an abnormal caudal vertebrae shape (Fig. 2).

Her karyotype was normal. Similarly, no abnormality was noted in tests for growth hormone, IGF‐1, IGFBP‐3, serum calcium, serum phosphorus, thyroid-stimulating hormone, parathyroid hormone, 25-hydroxy-vitamin D, prolactin, cortisol, adrenocorticotropic hormone, glycosylated hemoglobin, or glucose tolerance. In addition, amino acid, organic acid, and acylcarnitine spectra in blood and urine samples were also normal. Electroencephalographic results showed generally normal background activity with sporadic spikes and slow wave complexes, as well as multiple spikes and slow wave complexes in the bilateral parietal, occipital, and posterior temporal regions during non-rapid-eye-movement sleep. However, according to her parents’ observations, there was no indication of epilepsy.

**Fig. 3:**
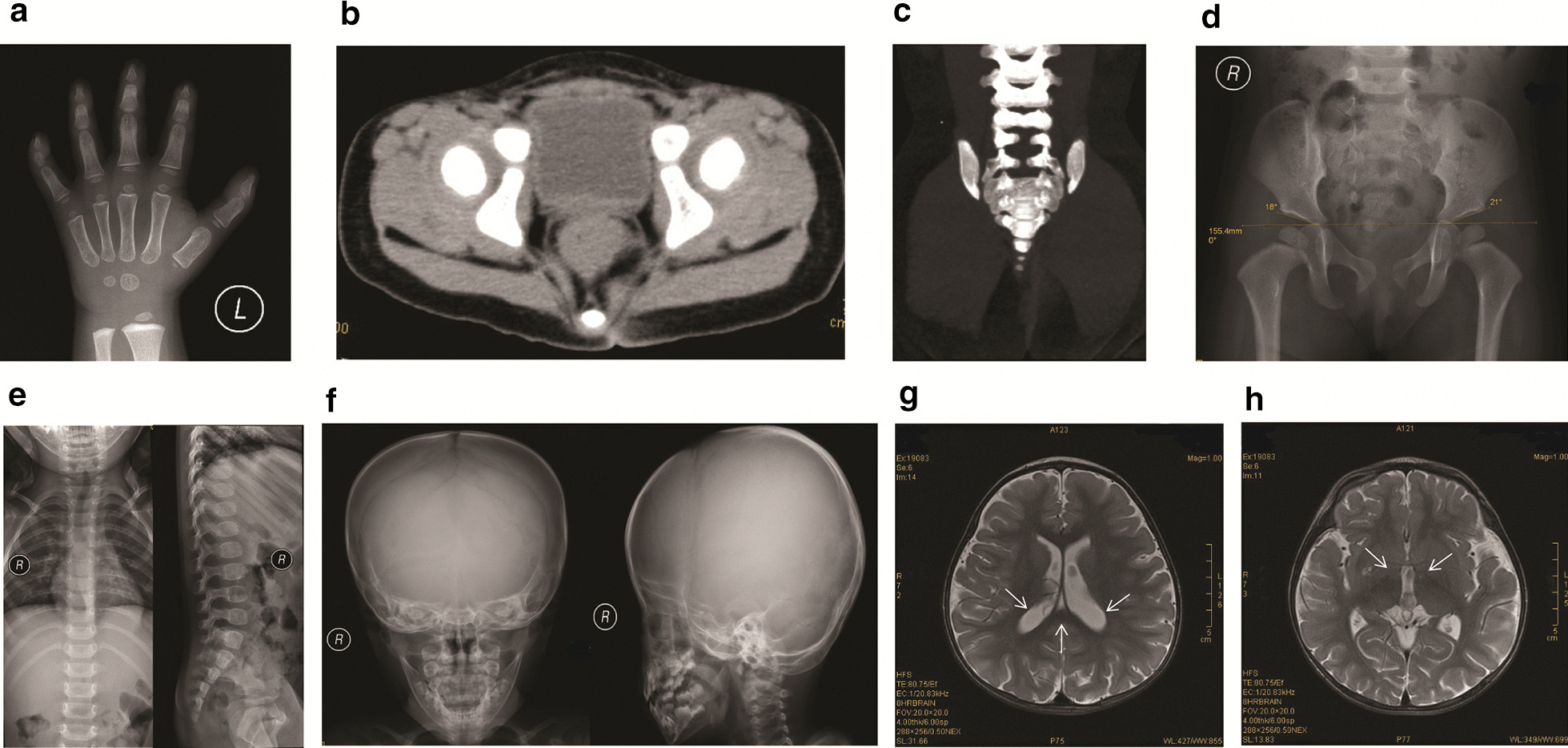
Imaging of the patient at the age of 2 years and 2 months. **a** Clinodactyly fifth finger. **b** Left-skewed caudal vertebra noted by the axial computed tomography (CT) scan. **c** Left-skewed caudal vertebra noted by the two-dimensional CT reconstruction. No abnormalities were noted in hip (**d**), main body of spine (**e**), and the skull (**f**). **g** Distended changes in bi-lateral ventricles (**g**) and the third ventricle (**h**), and the malformation of the sixth ventricles (**g**) were noted with the axial T2-weighted imaging. Arrows indicated the bi-lateral ventricles and the sixth ventricles in (**g**) and the third ventricle in (**h**)

Ultrasonic examinations showed no significant findings in the heart, liver, gallbladder, pancreas, spleen, adrenal glands, uterus, and ovaries. Radiographic examinations revealed delayed bone age (by approximately 8 months) and confirmed brachydactyly, fifth finger clinodactyly, and left-skewed sacral vertebrae (Fig. 3). Brain MRI showed a distended change in the bilateral ventricles and third ventricles, as well as malformation of the sixth ventricle (Fig. 3).

Beginning at the age of 4 months, the patient received two courses of physical therapy every three months including exercise training, low-frequency electrical stimulation, and water therapy. Beginning at the age of 24 months, the patient received individualized cognitive/linguistic training including eye contact, imitation training, linguistic expression, verbal comprehension, finger fine motor, and so on. In the meantime, she also received applied behavior analysis therapy with behavior analyst in the one-on-one manner. Neuropsychological evaluation was performed to patient at different ages. The evaluation was based on the Chinese version of child neuropsychological development checklist for ages 0–6 years [15]. Specifically, the checklist has 211 items including five categories (gross motor, fine motor, adaptive strengths, language, and social behaviors). Developmental quotient was used to indicate the results of neuropsychological evaluation. As shown in Table 1, no evident improvement was observed in the developmental delays after therapy, except for gross motor function.

## Genetic analysis

### Methods

Peripheral blood samples were collected from the patient and her parents. Genomic DNA was extracted using the QIAamp DNA Blood Mini Kit (Qiagen, Valencia, CA, USA) according to the manufacturer’s instructions. The DNA concentration was determined by measuring the absorbance at 260 nm using a NanoDrop 2000 spectrophotometer (Thermo Fisher, Waltham, MA, USA).

The DNA sample of the patient was analyzed by commercial whole-exome sequencing (WES, Fulgent Genetics, Fuzhou, Fujian, China). In short, the exome sequences from 1.0 μg of genomic DNA were enriched using a liquid capture system (Agilent SureSelect Human All Exon V5; Agilent Technologies, Santa Clara, CA, USA) according to the manufacturer’s protocol, and sequenced on the Hiseq4000 platform (Illumina, San Diego, CA, USA) to generate 150-bp paired-end reads. Detail information of WES results is listed in Additional file 1: Table S1.

The candidate variant was examined with Human Gene Mutation Database (HGMD, http://www.hgmd.cf.ac.uk), ClinVar database (https://www.ncbi.nlm.nih.gov/clinvar), dbSNP (https://www.ncbi.nlm.nih.gov/snp/), and gnomAD Browser (https://gnomad.broadinstitute.org/). The functional importance of the mutation was predicted using SIFT (http://provean.jcvi.org/index.php), PolyPhen-2 (http://genetics.bwh.harvard.edu/pph2/), and Mutation Taster databases (http://www.mutationtaster.org/).

### Results

**Fig. 4:**
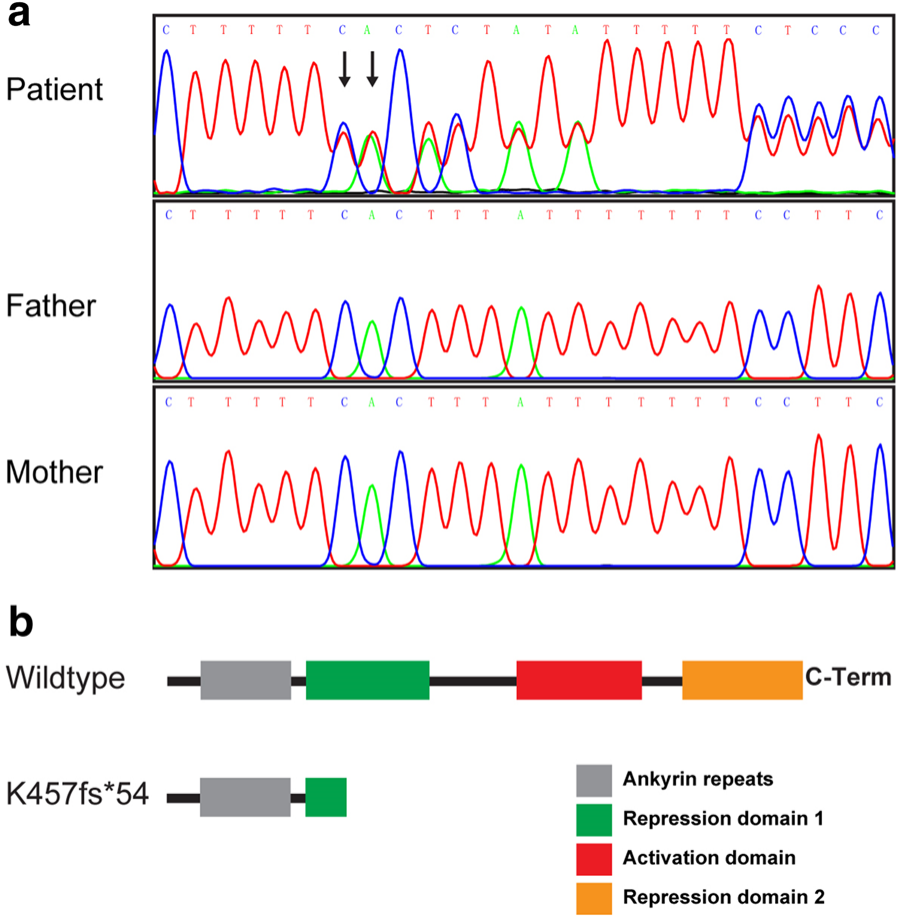
Nucleic acid and protein changes for c.1366_1367dup variant. **a** Sanger sequencing results of patient and her parents. Arrows indicates the duplicated nucleotides. **b** Predicted protein change for c.1366_1367dup variant

Among three WES-identified candidate variants (Additional file 1: Table S1), a novel heterozygous c.1366_1367dup (p.K457Rfs*54) variant in exon 9 of *ANKRD11* (NM_013275.6) was suggested as the target variant, and this variant was confirmed by subsequent Sanger sequencing. Moreover, Sanger sequencing revealed that the variant should be de novo since it was absent in the genomes of patient’s parents (Fig. 4). The variant was speculated to result in a truncated protein with a frameshift starting from the Lysine 457 (Fig. 4).

The *ANKRD11* c.1366_1367dup variant has not been previously reported and was absent in the HGMD, ClinVar, dbSNP, and gnomAD databases. Based on the SIFT (score: 0.007) and Polyphen-2 (score: 0.982 for HumDiv model and 0.952 for HumVar model, respectively) results, the p.K457Rfs*54 variant was predicted to be probably damaging to the structure/function of the protein. Similarly, based on the Mutation Taster results (probability: 1.000), this variant was predicted to be disease causing.

Eventually, the variant was interpreted as pathogenic according to the ACMG Standards and Guidelines (PVS1 + PS2 + PM2 + PP3 + PP4) [16].

## Discussion and conclusions

*ANKRD11* encodes ankyrin repeat domain-containing protein 11, which contains two transcriptional repression domains, a transcriptional activation domain, and an ankyrin repeat domain (Fig. 4) [13, 17]. ANKRD11 functions in transcriptional regulation by binding to chromatin-modifying enzymes, e.g., histone deacetylases. The protein is essential for the development and function of the nervous system and plays crucial roles in embryogenesis, skeletogenesis, and bone turnover [14, 18]. Heterozygous pathogenic variants in *ANKRD11* or deletions involving *ANKRD11* are the major causes of KBG syndrome [8]. To date, more than 300 variants in *ANKRD11* have been identified and deposited in the ClinVar database, including 104 frameshift, 193 missense, 63 nonsense, and 6 splicing variants (accessed on June 20, 2020). To our knowledge, the c.1366_1367dup variant in our case has not been reported previously. Similar to other frameshift variants identified in repression domain 1 (ClinVar database), in silico analysis predicted that the c.1366_1367dup variant is likely pathogenic. However, the explicit mechanism underlying the pathogenicity of *ANKRD11* variants in KBG syndrome remains unclear. Previous studies have suggested that variants leading to premature stop codons, especially in the N-terminal region, could trigger nonsense-mediated decay, resulting in haploinsufficiency [18, 19]. The findings for some variants that affect the N-terminal region of ANKRD11 suggest a dominant-negative effect could also be involved [19]. We speculated that haploinsufficiency was most likely responsible for the pathogenicity observed in our case due to nonsense-mediated decay or a non-functional truncated protein. However, further studies are needed to fully elucidate the mechanism underlying the pathogenicity of these *ANKRD11* variants in KBG syndrome.

Although accurate diagnosis of KBG syndrome relies on the identification of pathogenic changes in *ANKRD11*, early identification of suspect phenotypes is important, since it could direct the appropriate genetic evaluation and facilitate more effective prospective care of medical problems and genetic counseling. The phenotypic spectrum of KBG syndrome is wide, and there are no consensus clinical diagnostic criteria for KBG syndrome [20, 21]. Based on previous cases, the common phenotypes of KBG syndrome at an early age include craniofacial, skeletal, and neurologic abnormalities [22]. In terms of the associated craniofacial abnormalities, wide eyebrows, hypertelorism, a long philtrum, and macrodontia of the upper central incisors are frequently observed [8], and these were also present in our case (Fig. 2). However, compared to previous cases [8], the craniofacial abnormalities were mild in our case and was not a first clue to the diagnosis. In terms of skeletal anomalies, short stature is characteristic. In addition, delayed bone age, spinal anomalies, brachydactyly, and a large anterior fontanelle with delayed closure have been noted; these skeletal anomalies were also present in our case (Fig. 3). Specifically, brachydactyly and fifth finger clinodactyly were characteristic in our case and previous cases [8]. Moreover, caudal appendage, which was rare but specific in previous cases [8], was also identified in our case. These skeletal anomalies could be important clues leading to the further diagnosis. In terms of neurologic anomalies, developmental delays are characteristic. Seizures, various brain abnormalities, and behavioral issues have also been noted [8]. In our case, the dilation or malformation of several ventricles was observed (Fig. 3), suggesting the abnormal brain developments, which could result in the prominent developmental delay (Table 1) and isolated EEG-indicated abnormal electroencephalic activities. The *ANKRD11* might participate in the regulation of cellular mechanisms that underlie activity-dependent plasticity [13]. However, the underlying mechanism of various brain abnormalities induced with *ANKRD11* variants needs further investigation. Although seizures had not been noted, the EEG indicated abnormal electroencephalic activities, suggesting a close attention should be taken for potential seizures in the family care. Therefore, our case provides supportive evidence that combined presentations of short stature, developmental delay, and characteristic craniofacial and skeletal anomalies might be effective indicators for the genetic evaluation of KBG syndrome at an early age, and WES is one of the most valuable tools for genetic evaluation of clinically rare Mendelian diseases, owing to its low cost and high yield of clinical variants.

Current treatment of KBG syndrome depends on targeted management for its specific manifestations [20]. The patient in our case received physical therapy, individualized cognitive/linguistic training, and applied behavior analysis therapy at early age, and there was evident improvement in gross motor dysfunction (Table 1). Moreover, she would benefit from early physical therapy to maximize mobility and reduce the risk of later-onset orthopedic complications, such as scoliosis and hip dislocation. Growth hormone therapy and surgical correction will be performed to address the short stature and skeletal issues, respectively. Routine monitoring of hearing, vision, growth, and cognitive development are important for timely management of disease-associated conditions.

In conclusion, we reported a novel heterozygous frameshift *ANKRD11* c.1366_1367dup variant in a Chinese girl with KBG syndrome and the corresponding phenotypes. Our study expands the *ANKRD11* variant spectrum in KBG patients and provides clinical phenotypic data regarding KBG syndrome at an early age. Our study further demonstrates that WES is an effective method for the diagnosis of rare genetic disorders.

## Supplementary Information

**Additional file 1: **Candidates variants identified by whole exome sequencing.

## Data Availability

The raw data of whole-exome sequencing of the patient in this study are not publicly available in order to protect participant confidentiality but are available from the corresponding author on reasonable request. Reference sequence for *ANKRD11* (NC_000016.9) is available in the Genbank repository (https://www.ncbi.nlm.nih.gov/nuccore/NC_000016.9?report=genbank&from=89334038&to=89556969&strand=true). Databases used in this study were Human Gene Mutation Database (HGMD, http://www.hgmd.cf.ac.uk), ClinVar database (https://www.ncbi.nlm.nih.gov/clinvar), dbSNP (https://www.ncbi.nlm.nih.gov/snp/), gnomAD Browser (https://gnomad.broadinstitute.org/), SIFT (http://provean.jcvi.org/index.php), PolyPhen-2 (http://genetics.bwh.harvard.edu/pph2/), and Mutation Taster (http://www.mutationtaster.org/).

## Abbreviations

ANKRD11: Ankyrin repeat domain-containing protein 11
WES: Whole-exome sequencing
