# Supplementary material for: A de novo frameshift variant of ANKRD11 (c.1366_1367dup) in a Chinese patient with KBG syndrome
Source: BMC Med Genomics. 2021 Mar 2;14:68. doi: 10.1186/s12920-021-00920-3 (PMC7927266; doi:10.1186/s12920-021-00920-3)
Supplement: Supplementary file 1 — Additional file 1: Candidates variants identified by whole exome sequencing. [file 12920_2021_920_MOESM1_ESM.docx]

**Supplemental Table 1. Candidates variants identified by whole exome sequencing.**

| **Gene**  **(Reference transcript)** | **Variants** | **Homozygote/heterozygote** | **Inheritance** | **Classification** | **Associated phenotypes** | **Differential diagnosis** |
| --- | --- | --- | --- | --- | --- | --- |
| *ANKRD11*  (NM_013275.5) | c.1366_1367dup/p.Lys457Argfs*54 | Heterozygote | AD | Pathogenic | KBG syndrome  (MIM:148050 ) | The phenotypes of our case are in accordance with those of KBG syndrome |
| *PACS1*  (NM_018026.3) | c.1313A>G/p.Glu438Gly | Heterozygote | AD | Undetermined significance | Schuurs-Hoeijmakers syndrome  (MIM:615009 ) | Characteristic craniofacial abnormalities and hypotonia in this syndrome are not found in our case. Moreover, short stature in our case is not associated with this syndrome. |
| *NALCN*  (NM_001350748.1) | c.2692G>A/p.Ala898Thr | Heterozygote | AD | Undetermined significance | Congenital Contractures of the Limbs and Face, Hypotonia, and Developmental Delay  (MIM:616266) | Characteristic craniofacial abnormalities, hypotonia, and skeletal limbs abnormalities in this syndrome are not found in our case. Moreover, short stature in our case is not associated with this syndrome. |

The mean sequencing depth was 162.6×. Average coverage for exome was 98.4% at 30× of sequencing depth.
